# Effects of intravenous iron monotherapy for patients with iron deficient anemia undergoing total knee arthroplasty

**DOI:** 10.1186/s42836-020-00041-9

**Published:** 2020-08-03

**Authors:** Kyun-Ho Shin, Jong-Hoon Park, Ki-Mo Jang, Seok-Ha Hong, Seung-Beom Han

**Affiliations:** Department of Orthopedic Surgery, Anam Hospital, Korea University College of Medicine, 73 Inchon-ro, Sungbuk-gu, Seoul, 02841 South Korea

**Keywords:** Anemia, Iron deficiency, Iron, Ferric carboxymaltose, Transfusion, Total knee arthroplasty

## Abstract

**Background:**

Unnecessary costs and complications can be reduced by increasing hemoglobin (Hb) levels and minimizing blood transfusions in patients who underwent total knee arthroplasty (TKA). This study aimed to determine the effects of intravenous iron monotherapy before TKA on preoperative iron deficient anemia and postoperative transfusion rates.

**Methods:**

This prospective cohort study included 45 patients scheduled for TKA in the experimental group (Group I) and 221 patients who underwent TKA in 2015 and 2018 in the control group (Group C). One thousand milligrams of ferric carboxymaltose was administrated 1 month before TKA in group I. Intergroup comparison of the rate and total volume of transfusion, perioperative changes in Hb and analysis of iron metabolism variables in group I were performed. Subgroup analysis of Group I was conducted according to the response to iron monotherapy.

**Results:**

Although Hb levels increased after intravenous iron monotherapy in Group I, postoperative transfusion rates in Groups I and C were 17.8% and 18.6%, respectively, without significant intergroup differences in the rate and total units of transfusion. Ferritin level and transferrin saturation were corrected in both subgroups of Group I. Only 17 patients (37.78%) showed response to iron monotherapy, with an Hb increase of 1.0 g/dL or more. Subgroup analysis showed lower proportions of coexisting chronic diseases, including chronic kidney disease in responders.

**Conclusion:**

IV iron monotherapy was shown to be insufficient in successfully treating preoperative iron-deficient anemia to reduce postoperative allogenic blood transfusion in patients who underwent TKA. As preoperative anemia should be managed due to the high rates of postoperative transfusion for this surgery, clinicians should consider the complex interplay among the causal factors of anemia, apart from ID, in patients with preoperative anemia who are scheduled for TKA.

## Background

Total knee arthroplasty (TKA) is one of the most common treatment procedures for advanced osteoarthritis of the knee joint, and more than 600,000 TKA surgeries are performed annually in the United States [1]. The procedure is associated with substantial blood loss and frequent use of allogenic blood transfusions to manage the occurrence of perioperative anemia, which is associated with potential risks and complications, such as periprosthetic joint infection, disease transmission, fluid overload, cardiac arrhythmia, immunologic reactions, and even death [2–5]. Hemoglobin (Hb) levels should be increased and perioperative blood transfusions minimized in patients who underwent TKA in order to avoid unnecessary expenses for the health care system.

Preoperative anemia is considered a common factor leading to postoperative blood transfusions, and iron deficiency (ID) is the most common cause of anemia, with a prevalence rate of 41% [6]. Several blood management strategies, such as erythropoietin administration and intravenous (IV) or oral iron supplementation, have been used to achieve optimal preoperative Hb levels and reduce the requirement of postoperative transfusion [7, 8]. Studies have indicated that iron supplementation is a safe and efficient method for increasing preoperative Hb levels prior to major orthopedic surgeries [9–11]. However, the effects and role of IV iron monotherapy in patients with preoperative iron deficient anemia who undergo TKA remain unclear.

We hypothesized that IV iron monotherapy would normalize Hb levels and reduce transfusion rates in patients with preoperative iron deficient anemia scheduled to undergo TKA. This study aimed to determine the effectiveness of IV iron monotherapy in a clinical setting.

## Methods

### Patients

The study protocol was approved by the Institutional Review Board of our institution, and informed consent was obtained from all patients in the experimental group. This single-center prospective cohort study enrolled 46 patients into the experimental group (Group I) who were scheduled to undergo unilateral TKA for primary osteoarthritis of the knee joint between January 2017 and January 2019. The inclusion criteria were as follows: (1) preoperative anemia, defined as Hb levels < 12.0 g/dL for women and < 13.0 g/dL for men according to the World Health Organization classification, and (2) ID, defined as a ferritin concentration < 30 μg/L or 30–100 μg/L and transferrin saturation (TSAT) < 20%. Exclusion criteria were macrocytic anemia which was defined as a mean corpuscular volume over 100 fL, abnormal liver function, hypersensitivity to ferric carboxymaltose, iron supplementation, and history of previous blood transfusion within 1 month before operation. No case was excluded in Group I against the exclusion criteria.

For the control group, we reviewed the data collected from 937 consecutive primary TKA procedures for primary osteoarthritis performed between 2015 and 2018. The inclusion criterion was preoperative anemia except macrocytic anemia. Anemia was treated with IV iron preoperatively in patients undergoing bilateral TKA procedures and at the interval period in those undergoing staged TKA procedures with an interval of less than 3 months.

**Fig. 1:**
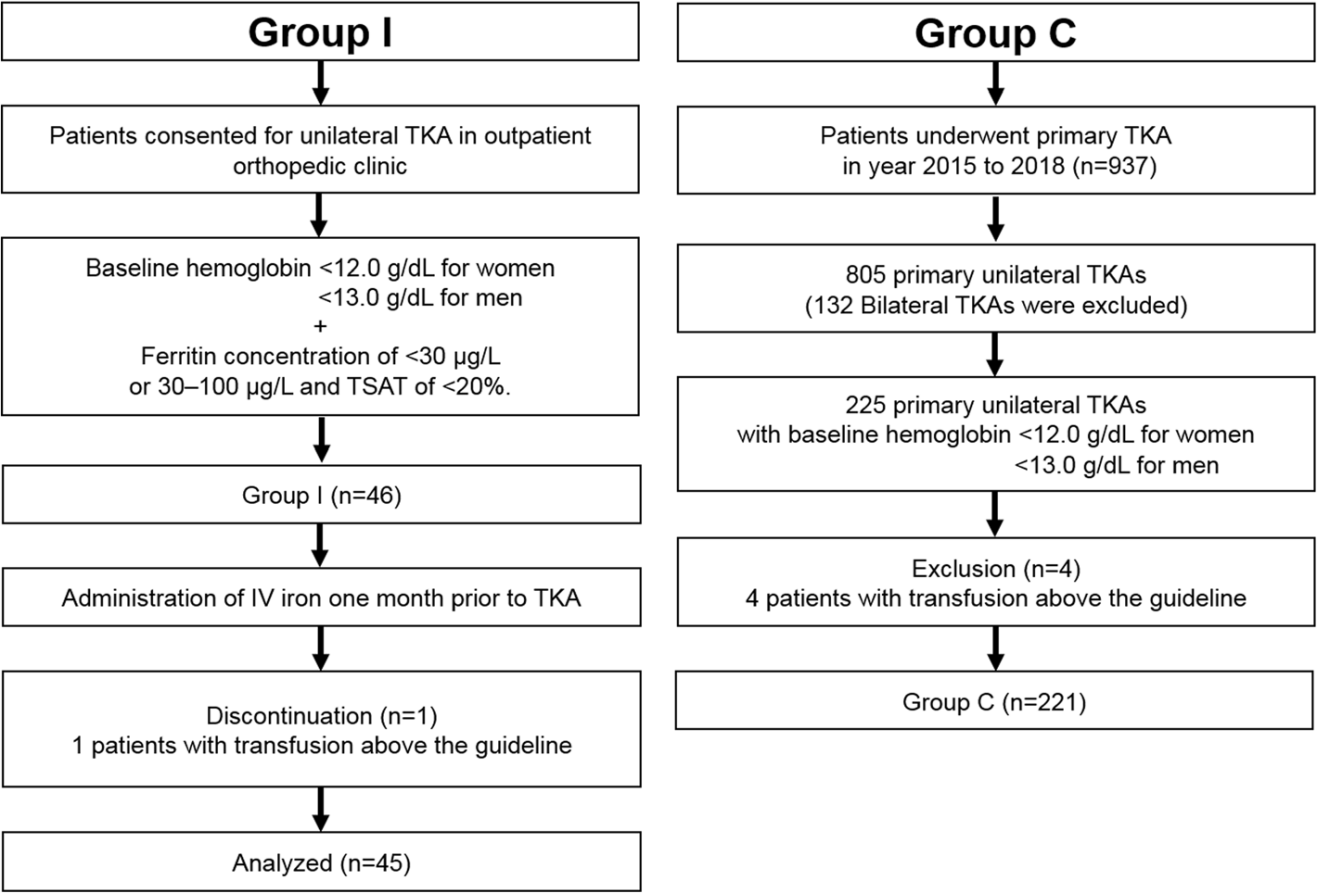
Flowchart of patient enrolment

As a result, patients who underwent simultaneous bilateral TKA procedures were excluded. In staged cases in which TKA was performed bilaterally with an interval of at least 1 week to 3 months, the side on which TKA was performed first was selected. In staged cases in which TKA was performed bilaterally with an interval of more than 3 months, both sides were selected. Of the 937 consecutive primary TKAs, 132 simultaneous bilateral TKA procedures were excluded. Among 805 primary TKAs, 225 primary TKAs performed in 225 patients with preoperative anemia were finally enrolled into the control group (Group C). The flowchart of patient selection is summarized in Fig. 1.

### Iron administration and surgical technique

Iron was administered via IV injection. Group I participants were administered IV iron 1 month preoperatively in an outpatient clinic. Ferric carboxymaltose (1000 mg, FERINJECT INJ; Vifor International Ltd., Glattbrugg, Switzerland) was administered intravenously over a 1-h period. Because an increase in Hb levels was achieved ≥2 weeks after IV iron administration, Group I patients received IV iron monotherapy approximately 4 weeks preoperatively [12].

All TKA procedures were performed by the same surgical team using a cemented posterior cruciate-substituting design and resurfacing of the patella. The following two types of prosthesis were used in the operation: e.motion® Total Knee System (B. Braun Aesculap, Tuttlingen, Germany) and Attune (Depuy Synthes, Warsaw, IN). The type of anesthetic administered (spinal or general) was decided by the anesthesiologist and the patient involved. The surgical procedures were performed using the medial parapatellar approach with patellar eversion under pneumatic tourniquet inflation. Electrocautery was performed to achieve hemostasis throughout the procedure, and wounds were closed using a non-invasive secure skin closure (Zip 16 Surgical Skin Closure System, Zipline Medical, Campbell, CA) after subcutaneous tissue closure. Closure was routinely performed with the knee in the flexed position and antifibrinolytics were not used. Closed suction drains (Hemovac, Zimmer, Inc., Warsaw, IN, USA) were placed at the knee joint after the completion of the main procedure and were removed after 48 h. Early mobilization and continuous passive motion were implemented after removal of the drains. Prophylaxis against venous thromboembolism was performed using low molecular weight heparin (Clexane; Sanofi, Paris, France) for 1 week after the surgery.

In all participants, the red blood cell (RBC) transfusion guidelines were applied as follows: 1 unit of RBCs (derived from 400 mL of whole blood) was transfused to participants with Hb levels of 7.0–7.9 g/dL, whereas 2 units of RBCs were transfused to those with Hb levels of 6.0–6.9 g/dL. Transfusions were immediately performed after patients developed symptoms of anemia and hypovolemia, including hypotension, tachycardia, tachypnea, and dizziness. Five patients (one in Group I and four in Group C) who underwent emergency transfusions were excluded from the study.

### Perioperative laboratory and clinical data

Baseline Hb and hematocrit (Hct) values, and platelet counts were measured 1 month preoperatively in both groups. Additional blood samples were drawn immediately before the surgery to obtain Hb values in Group I. Postoperatively, Hb and Hct levels were measured at day 1, day 3 and day 7 in both groups. In group I, iron metabolism indices, such as iron and ferritin serum concentrations and TSAT were measured 1 month preoperatively, immediately before the surgery, and 1 and 7 day(s) after operation. The total volume of postoperative blood drainage was calculated. Total blood loss was estimated using Mercuriali’s formula, which is reliable for the comparison of blood loss in TKA [13, 14]. Since the total blood loss calculated using the Mercuriali’s formula is expressed in milliliters of red blood cells (RBCs), conversion to milliliters of blood was performed using the average of the preoperative and final hematocrit volumes. Other clinical outcomes (amount of postoperative bleeding measured through the closed suction drainage, transfusion rate, and total volume of transfused RBCs) were recorded daily, from the day of surgery to postoperative day 7. Iron status was assessed only in Group I.

Primary outcomes were the transfusion rate and total volume of transfused RBCs. Secondary outcome was perioperative changes of Hb levels after iron administration. Responders were defined as subjects with an Hb increase of 1.0 g/dL or more immediately before the surgery and non-responders were subjects with an Hb increase of less than 1.0 g/dL. For further analysis, Group I was subdivided into two subgroups in terms of the response to iron monotherapy, i.e., 17 responders and 28 non-responders. The baseline and perioperative characteristics and perioperative changes in the Hb level and iron metabolism indices between the two subgroups were analyzed.

### Statistical analysis

To determine the sample size of the study, a priori power analysis was performed using Hb level as the primary outcome variable. A previous report indicated the optimization of preoperative Hb level through IV iron administration ranged from 11.9 ± 0.8 to 12.7 ± 0.7 g/dL [12]. A minimum sample size of 27 patients in the experimental group and 55 in the control group (82 total patients) was estimated to provide 80% power to detect intergroup differences of this magnitude (G*Power, version 3.1.3; www.gpower.hhu.de). The sample size in the experimental group was increased to 46 patients to account for potential dropouts. All data are presented as mean and standard deviation for continuous variables and as number and percentage for categorical variables. The demographic characteristics and perioperative data of patients in the two groups were compared using the Student’s *t* test, the Mann-Whitney U-test and the Fisher’s exact test. Differences between Hb levels and iron status during the perioperative days were analyzed using repeated-measures analysis of variance. A Bonferroni *post-hoc* test was conducted to compare statistically significant differences between Hb levels and iron status at each time point. Simple and multiple Logistic regression analyses were performed to evaluate the effect of iron monotherapy on transfusion rate. The multiple Logistic regression model was adjusted for all covariates with a *P*-value < 0.1 in the univariate analysis.

The propensity scores (PS) were estimated using multiple Logistic regression analyses, with the administration of intravenous iron as the dependent variable. All parameters in Table 1 except estimated blood loss and postoperative drainage were used to determine the propensity scores. To reduce the effects of possible confounding factors in the two groups, we performed PS matching analysis. The two groups were well matched in all parameters (Table 1).

**Table 1:** Baseline and perioperative characteristics of the two groups

Variables	Crude		***p***-value	***PS Matching***		***p***-value
	Group I (***n*** = 45)	Group C (***N*** = 221)		Group I (***n*** = 45)	Group C (***n*** = 45)	
Age (y)	67.71 ± 7.94	71.96 ± 6.19	< 0.001	67.71 ± 7.94	69.82 ± 5.38	0.143
Sex (F/M)	40 (88.9%)/5 (11.1%)	203 (91.9%)/18 (8.1%)	0.519	40 (88.9%)/5 (11.1%)	40 (88.9%)/5 (11.1%)	1.000
Body weight (kg)	57.10 ± 4.97	59.96 ± 7.35	0.013	57.10 ± 4.97	56.84 ± 6.71	0.838
Hypertension	30 (66.7%)	152 (68.8%)	0.781	30 (66.7%)	31 (68.9%)	0.822
Diabetes	13 (28.9%)	65 (29.4%)	0.944	13 (28.9%)	13 (28.9%)	1.000
Chronic kidney disease	7 (15.6%)	50 (22.6%)	0.292	7 (15.6%)	8 (17.8%)	0.777
Cerebrovascular disease	6 (13.3%)	29 (13.1%)	0.970	6 (13.3%)	6 (13.3%)	1.000
Cardiovascular disease	9 (20.0%)	56 (25.3%)	0.447	9 (20.0%)	10 (22.2%)	0.796
Thyroid disease	5 (11.1%)	21 (9.5%)	0.740	5 (11.1%)	5 (11.1%)	1.000
Antiplatelets	12 (26.7%)	834 (37.6%)	0.165	12 (26.7%)	15 (33.3%)	0.490
Anticoagulants	1 (2.2%)	7 (3.2%)	0.735	1 (2.2%)	1 (2.2%)	1.000
Baseline hemoglobin (g/dL)	11.41 ± 1.12	11.46 ± 1.01	0.750	11.41 ± 1.12	11.40 ± 0.93	0.951
Preoperative platelet counts (× 10^9^/L)	267.56 ± 72.33	249.21 ± 60.88	0.076	267.56 ± 72.33	253.29 ± 59.81	0.311
Estimateld blood loss (mL)	519.90 ± 145.55	514.25 ± 199.52	0.825	519.90 ± 145.55	531.54 ± 183.87	0.740
Postoperative drainage (mL)	317.40 ± 157.59	285.86 ± 135.82	0.169	317.40 ± 157.59	288.86 ± 137.05	0.352

Data were analyzed using the Statistical Package for the Social Sciences version 22.0 software (IBM Corp., Armonk, NY, USA), and differences were considered significant at *P* < 0.05.

## Results

There was no intergroup difference in either the baseline characteristics or perioperative findings of the patients after PS matching (Table 1). There were no wound complications in both two groups.

The primary and secondary outcomes are listed in Tables 2 and 3. Differences in the rate and total volume of transfusion were not significant between Groups I and C (Table 2). Eight patients (17.8%) in Group I and 14 (18.6%) in Group C were transfused with RBCs (Odds ratio [OR] = 9.949, *p* = 0.903). There was no significant reduction in the rate of transfusion even after PS matching analysis (OR = 0.754, *p* = 0.754). Furthermore, there was no intergroup difference in total volume of transfusion before and after PS matching analysis.

**Table 2:** Comparison of rates and total volume of transfusion between two groups

Outcome	Group	Crude			PS matching		
		Event/*n*	OR (95% CI)	***p***-value	Event/*n*	OR (95% CI)	***p***-value
Rate of transfusion	Group I	8/45	0.949 (0.411, 2.190)	0.903^a^	8/45	0.754 (0.212, 2.686)	0.754^b^
	Group C	41/221			9/45		
		**Mean ± SD**		***p*-value**	**Mean ± SD**		***p*-value**
Volume of transfusion (mL)	Group I	35.56 ± 77.33		0.602^c^	35.56 ± 77.33		0.791^c^
	Group C	43.44 ± 94.94			40.00 ± 80.90		

*PS* propensity score

^a^*p*-value by simple Logistic regression; ^b^*p*-value by conditional Logistic regression with adjusted variables in Table 1, ^c^*p*-value by student’s *t* test

**Table 3:** Comparison of perioperative changes in mean hemoglobin levels (g/dL) between Groups I and C

	Group	Baseline	Preoperative	Postoperative day 1	Postoperative day 3	Postoperative 1 week
**Crude**	**Group I**	11.41 ± 1.12	12.14 ± 1.15	11.32 ± 0.92	10.42 ± 0.96	9.06 ± 0.84
	**Group C**	11.46 ± 1.00	…	10.59 ± 1.15	9.81 ± 1.04	8.88 ± 0.91
	*p*-value^a^	0.750	**0.001**^**b**^	**< 0.001**	**< 0.001**	0.272
**PS matching**	**Group I**	11.41 ± 1.12	12.14 ± 1.15	11.32 ± 0.92	10.42 ± 0.96	9.06 ± 0.84
	**Group C**	11.40 ± 0.93	…	10.40 ± 1.10	9.85 ± 1.13	8.85 ± 0.77
	*p*-value^a^	0.951	**0.001**^**b**^	**< 0.001**	**0.012**	0.241

*PS* propensity score

^a^Comparison between two groups using student’s *t* test

^b^Comparison between baseline and preoperative hemoglobin levels of Group I using paired *t*-test

Perioperative serum Hb levels are shown in Table 3. Serum Hb levels, after an IV iron monotherapy, were significantly increased in Group I, with the difference being 0.727 g/dL. Similar perioperative changes in serum Hb level were observed in both groups. Postoperative serum Hb levels were significantly decreased during the first postoperative week (Table 3). Hb level was significantly different between the two groups 1 and 3 day(s) after operation, while no difference in Hb level was shown at day 7 postoperatively (Table 3).

Measurements of iron metabolism variables in Group I and its subgroups are shown in Table 4. After an IV administration of iron supplement, the mean ferritin level increased from 41.39 to 866.73 μg/L and TSAT was corrected by 25.46% in Group I. Also, the ferritin level rose after the IV administration of iron supplementation in Group I. TSAT increased after the IV iron supplementation and no significant TSAT changes were observed during the postoperative period. As to the adverse effects of iron supplementation, one patient experienced mild myalgia in both lower extremities for several days. No other complications were observed in this study.

**Table 4:** Iron metabolism variables of Group I

		Baseline (1)	Preoperative (2)	Postoperative 1 day (3)	Postoperative 1 week (4)	***p***-value^a^
Ferritin (μg/L)	Mean ± SD	41.39 ± 24.44	866.73 ± 327.27	890.56 ± 328.14	739.63 ± 366.24	**< 0.001**
	*Post-hocp*-value	1vs2, **< 0.001**	1*vs*. 3, **< 0.001**	1 *vs*. 4, **< 0.001**	2 *vs*. 3, 1.000	
		2v4, 0.192	3 *vs*. 4, 0.080			
Serum iron (g/dL)	Mean ± SD	39.59 ± 18.08	68.38 ± 25.90	71.22 ± 32.24	48.91 ± 11.08	**< 0.001**
	*Post-hocp*-value	1 *vs*. 2, **< 0.001**	1 *vs*. 3, **< 0.001**	1 *vs*. 4, **0.013**	2 *vs*. 3, 1.000	
		2 *vs*. 4, **< 0.001**	3 *vs*. 4, **< 0.001**			
TSAT (%)	Mean ± SD	11.87 ± 4.09	25.46 ± 9.03	26.19 ± 10.30	24.80 ± 8.46	**< 0.001**
	*Post-hocp*-value	1 *vs*. 2, **< 0.001**	1 *vs*. 3, **< 0.001**	1 *vs*. 4, **< 0.001**	2 *vs*. 3, 1.000	
		2 *vs*. 4, 1.000	3 *vs*. 4, 1.000			

TSAT, transferrin saturation; SD, standard deviation

^a^Comparison between two groups using the Repeated-measures analysis of variance test

Results of subgroup analyses according to the response to the iron monotherapy are shown in Supplement tables 1 and 2. There was significant intergroup difference in the proportion of chronic kidney disease (Supplement table 1). Despite no statistically significant differences, the rate of transfusion and proportion of other coexisting morbidities, including cardiovascular and cerebrovascular diseases were lower in responders (Supplement table 1). After an IV iron supplement administration, TSAT was corrected in both subgroups of Group I (Supplement table 2).

## Discussion

This study revealed that IV iron monotherapy before TKA did not affect the rate or total units of transfusion in patients with preoperative anemia. Although there was significant increase in the mean Hb level after iron monotherapy, the difference was only 0.727 g/dL and it did not reduce the rate of transfusion. Despite the restricted transfusion trigger, both Groups I and C achieved relatively high rates of allogenic blood transfusion of 17.8% (8/45) and 18.6% (41/221), respectively.

TKA is generally associated with high rates of allogenic blood transfusion due to substantial blood loss during and after operation. However, allogenic blood transfusions pose several potential risks and cause complications when used to treat perioperative anemia [2–5]. Previous studies have reported that 10.8% of patients scheduled for elective hip and knee arthroplasty have preoperative anemia, of which ID is the most common cause (prevalence of 41%) [6]. Considering that preoperative anemia is an indicator for postoperative blood transfusions, clinicians should increase patients’ Hb levels to minimize blood transfusions associated with TKA [4].

The positive clinical effectiveness of perioperative iron therapy in various surgeries, including the cardiac surgery, major orthopedic surgeries, has been reported [15, 16]. Similar to our study regarding iron therapy for ID anemia, the research by Theusinger *et al* reported that IV iron administration could effectively correct ID anemia before elective orthopedic surgeries [12]. However, allogenic blood transfusion rates remained unimproved although Hb levels improved after IV iron monotherapy 4 weeks prior to their surgery in Group I, and mean increase in Hb levels did not suffice to affect the rate or total volume of transfusion in group I. Only 17 patients (37.78%) were responders and showed increased Hb levels along with ID correction. On the contrary, the remaining 28 non-responders (62.42%) in group I showed no changes in Hb levels, which remained low despite the correction of ID. This may be because anemia is mainly caused by ID or a complex interplay among chronic inflammation, ID, and renal impairment in the elderly population [17, 18]. Administration of IV iron therapy without erythropoietin can improve Hb levels, even in patients with chronic kidney disease. However, erythropoiesis-stimulating agent (ESA) is typically administered to treat anemia in patients with end-stage kidney disease and those with inflammatory/chronic diseases who are scheduled for elective surgery [19–21]. In the present study, the incidence of chronic kidney disease was high in Groups I and C (15.6 and 22.6%, respectively). Furthermore, 17 responders in the subgroup with ID did not present with chronic kidney disease, chronic inflammation, or cancer, whereas 6 of 28 non-responders presented higher proportion of chronic kidney disease and other chronic diseases, including cardio- and cerebro-vascular disorders. This finding highlights that a heterogeneous interplay of several diseases (regardless of ID) may cause preoperative anemia and might be responsible for the lack of a significant relationship between IV iron monotherapy and iron deficient anemia to reduce blood transfusion rates in our patients. In other words, IV iron monotherapy might have some advantages in selected patients with iron-deficient anemia and without other chronic diseases for reduction of transfusion rates. However, the proportion of such patients undergoing TKA is very lower and the identification and management of not only ID but also other accompanying chronic diseases should be considered to reduce the postoperative transfusion rate in patients with preoperative anemia undergoing TKA.

The strength of this study lies in its exact and precise analysis of outcomes, which was possible with matching of baseline confounders by PS matching analysis. To the best of our knowledge, our study is the first matched study to compare the effectiveness of iron monotherapy for patients with preoperative iron-deficient anemia who underwent TKA. The focus of this study was to raise awareness of the cost-effectiveness of iron monotherapy for patients with iron-deficient anemia in TKA. Clinicians should consider the fact that IV iron monotherapy may not be an ideal method for patients with other causal factors of anemia, such as chronic inflammation or renal impairment, to reduce the transfusion rate and successfully treat preoperative anemia in patients scheduled for TKA. Preoperative anemia should be diagnosed to administer proper management in order to mitigate the risk of perioperative transfusions during TKA due to the high rate of allogenic blood transfusion in the present study. Further studies on different treatments of preoperative anemia according to cause, individualized blood management strategies, or combined ESA and iron supplementation are needed to increase preoperative Hb levels and reduce transfusion rates.

Our study had several limitations. First, the study was conducted at a single institution with a relatively small number of patients. Despite the prospective cohort study design, randomization was not performed due to limited adequate placebo treatments during the study period. Second, the iron status was not assessed in the control group. Third, other various causes of anemia, such as chronic inflammation, chronic kidney disease, and cancer, were not controlled. However, the selected proportion of patients without chronic diseases are very lower when the actual clinical practice is reflected. Furthermore, we used PS matching analysis to adjust the effects of the confounding factors and reduce bias. Multi-center, large-scale and randomized studies might be needed to compare the effectiveness between iron monotherapy and individualized blood management strategies according to the various causes of anemia. Although no definite conclusions could be made regarding the use of IV iron monotherapy, our results provided preliminary data for the development of a clinical approach aimed at reducing the rate of allogenic blood transfusions and treating anemia, in addition to verifying the clinical effects of IV iron monotherapy for patients with preoperative iron-deficient anemia in TKA.

## Conclusion

IV iron monotherapy was shown to be insufficient in successfully treating preoperative iron-deficient anemia to reduce postoperative allogenic blood transfusion in patients who underwent TKA. As preoperative anemia should be managed due to the high rates of postoperative transfusion for this surgery, clinicians should consider the complex interplay among the causal factors of anemia, apart from ID, in patients with preoperative anemia who are scheduled for TKA.

## Supplementary information

**Additional file 1: Supplement 1.** Comparison of baseline and perioperative characteristics between responders and non-responders.

**Additional file 2: Supplement 2.** Comparison of iron metabolism variables between responders and non-respondoners in group I.

## Data Availability

Data sharing is not applicable to this article as no datasets were generated or analysed during the current study.

## Abbreviations

ESA: Erythropoiesis-stimulating agent
Hb: Hemoglobin
Hct: Hematocrit
ID: Iron deficiency
IV: Intravenous
PS: Propensity score
RBC: Red blood cell
TKA: Total knee arthroplasty
TSAT: Transferrin saturation

## Supplementary information

**Supplementary information** accompaniying this paper is at 10.1186/s42836-020-00041-9.

## References

[1] Melvin JS, Stryker LS, Sierra RJ (2015). Tranexamic acid in hip and knee arthroplasty. J Am Acad Orthop Surg.

[2] Friedman R, Homering M, Holberg G, Berkowitz SD (2014). Allogeneic blood transfusions and postoperative infections after total hip or knee arthroplasty. J Bone Joint Surg Am.

[3] Klika AK, Small TJ, Saleh A, Szubski CR, Chandran Pillai AL, Barsoum WK (2014). Primary total knee arthroplasty allogenic transfusion trends, length of stay, and complications: nationwide inpatient sample 2000-2009. J Arthroplast.

[4] Maempel JF, Wickramasinghe NR, Clement ND, Brenkel IJ, Walmsley PJ (2016). The pre-operative levels of hemoglobin in the blood can be used to predict the risk of allogenic blood transfusion after total knee arthroplasty. Bone Joint J.

[5] Ponnusamy KE, Kim TJ, Khanuja HS (2014). Perioperative blood transfusions in orthopedic surgery. J Bone Joint Surg Am.

[6] Jans O, Nielsen CS, Khan N, Gromov K, Troelsen A, Husted H (2018). Iron deficiency and preoperative anemia in patients scheduled for elective hip- and knee arthroplasty—an observational study. Vox Sang.

[7] Dragan S, Kulej M, Krawczyk A, Wall A, Płocieniak K, Urbański W (2012). Methods of reducing allogeneic blood demand in orthopedic surgery. Ortop Traumatol Rehabil.

[8] Marti-Carvajal AJ, Agreda-Perez LH, Sola I, Simancas-Racines D (2013). Erythropoiesis-stimulating agents for anemia in rheumatoid arthritis. Cochrane Database Syst Rev.

[9] Avni T, Bieber A, Grossman A, Green H, Leibovici L, Gafter-Gvili A (2015). The safety of intravenous iron preparations: systematic review and meta-analysis. Mayo Clin Proc.

[10] Cuenca J, Garcia-Erce JA, Martinez F, Pérez-Serrano L, Herrera A, Muñoz M (2006). Perioperative intravenous iron, with or without erythropoietin, plus restrictive transfusion protocol reduce the need for allogeneic blood after knee replacement surgery. Transfusion.

[11] Munoz M, Gomez-Ramirez S, Cuenca J, García-Erce JA, Iglesias-Aparicio D, Haman-Alcober S (2014). Very-short-term perioperative intravenous iron administration and postoperative outcome in major orthopedic surgery: a pooled analysis of observational data from 2547 patients. Transfusion.

[12] Theusinger OM, Leyvraz PF, Schanz U, Seifert B, Spahn DR (2007). Treatment of iron deficiency anemia in orthopedic surgery with intravenous iron: efficacy and limits: a prospective study. Anesthesiology.

[13] Mercuriali F, Inghilleri G (1996). Proposal of an algorithm to help the choice of the best transfusion strategy. Curr Med Res Opin.

[14] Gibon E, Courpied JP, Hamadouche M (2013). Total joint replacement and blood loss: what is the best equation?. Int Orthop.

[15] Muñoz M, Gómez-Ramírez S, Cuenca J, García-Erce JA, Iglesias-Aparicio D, Haman-Alcober S, Ariza D, Naveira E (2014). Iron and postoperative outcome in orthopedic surgery. Transfusion.

[16] Torres S, Kuo YH, Morris K, Neibart R, Holtz JB, Davis JM (2006). Intravenous iron following cardiac surgery does not increase the infection rate. Surg Infect.

[17] Bach V, Schruckmayer G, Sam I, Kemmler G, Stauder R (2014). Prevalence and possible causes of anemia in the elderly: a cross-sectional analysis of a large European university hospital cohort. Clin Interv Aging.

[18] Goodnough LT, Schrier SL (2014). Evaluation and management of anemia in the elderly. Am J Hematol.

[19] Muñoz M, Martín-Montañez E (2012). Ferric carboxymaltose for the treatment of iron-deficiency anemia. Expert Opin Pharmacother.

[20] KDIGO (2012). Clinical practice guideline for Anaemia in chronic kidney disease. Kidney Int Suppl.

[21] Goodnough LT, Shander A (2013). Current status of pharmacologic therapies in patient blood management. Anesth Analg.

