# Supplementary material for: Effects of intravenous iron monotherapy for patients with iron deficient anemia undergoing total knee arthroplasty
Source: Arthroplasty. 2020 Aug 3;2:22. doi: 10.1186/s42836-020-00041-9 (PMC8796593; doi:10.1186/s42836-020-00041-9)
Supplement: Supplementary file 1 — Additional file 1: Supplement 1. Comparison of baseline and perioperative characteristics between responders and non-responders. [file 42836_2020_41_MOESM1_ESM.doc]

Supplement 1. Comparison of baseline and perioperative characteristics between responders and non-responders

| **Variables** | **Responder (N = 17)** | **Non-responder (N = 28)** | ***P*-value** |
| --- | --- | --- | --- |
| Age (y) | 67.24 ± 7.42 | 68.00 ± 8.36 | 0.758 |
| Sex (F/M) | 15 (88.2%)/2 (11.8%) | 25 (89.3%)/3 (10.7%) | 1.000 |
| Body weight (kg) | 57.29 ± 4.54 | 56.98 ± 5.29 | 0.841 |
| Hypertension | 10 (58.8%) | 20 (71.4%) | 0.517 |
| Diabetes | 5 (29.4%) | 8 (28.6%) | 1.000 |
| Chronic kidney disease | 0 (0%) | 7 (25.0%) | **0.034** |
| Cerebrovascular disease | 2 (11.8%) | 4 (14.3%) | 1.000 |
| Cardiovascular disease | 2 (11.8%) | 7 (25.0%) | 0.447 |
| Thyroid disease | 2 (11.8%) | 3 (10.7%) | 1.000 |
| Antiplatelets | 4 (23.5%) | 8 (28.6%) | 1.000 |
| Anticoagulants | 0 (0%) | 1 (3.6%) | 1.000 |
| Baseline Hb (g/dL) | 11.41 ± 1.12 | 11.46 ± 1.01 | 0.750 |
| Preoperative Hb (g/dL) | 12.98 ± 1.06 | 11.63 ± 0.88 | <0.001 |
| POD 1 Hb | 11.79 ± 0.78 | 11.04 ± 0.90 | 0.007 |
| POD 3 Hb | 10.75 ± 0.84 | 10.22 ± 0.99 | 0.075 |
| POD 7 Hb | 9.26 ± 0.71 | 8.93 ± 0.90 | 0.196 |
| Preoperative platelet counts (×10^9^/L) | 272.06 ± 90.45 | 264.82 ± 88.19 | 0.749 |
| Estimateld blood loss (mL) | 520.43 ± 166.38 | 519.57 ± 134.60 | 0.985 |
| Postoperative drainage (mL) | 314.76 ± 166.38 | 319.00 ± 170.85 | 0.932 |
| Rate of Transfusion | 1 (5.9%) | 7 (25.0%) | 0.132 |
| Total volume of transfusion | 11.76 ± 48.51 | 50.00 ± 88.19 | 0.068 |
