# Supplementary material for: Effects of intravenous iron monotherapy for patients with iron deficient anemia undergoing total knee arthroplasty
Source: Arthroplasty. 2020 Aug 3;2:22. doi: 10.1186/s42836-020-00041-9 (PMC8796593; doi:10.1186/s42836-020-00041-9)
Supplement: Supplementary file 2 — Additional file 2: Supplement 2. Comparison of iron metabolism variables between responders and non-respondoners in group I. [file 42836_2020_41_MOESM2_ESM.doc]

Supplement 2. Comparison of iron metabolism variables between responders and non-respondoners in group I

|  | **Group** | **Baseline** | **Preoperative** | **Postoperative 1 day** | **Postoperative 1 week** |
| --- | --- | --- | --- | --- | --- |
| Ferritin (μg/L) | Responder | 46.51 ± 27.35 | 955.00 ± 319.53 | 979.90 ± 250.27 | 694.43 ± 234.80 |
|  | Non-Responder | 38.29 ± 22.44 | 813.14 ± 325.82 | 836.31 ± 360.94 | 767.08 ± 428.78 |
|  | P-value | 0.535 | 0.292 | 0.120 | 0.991 |
| Serum iron  (g/dL) | Responder | 37.79 ± 18.51 | 75.91 ± 18.71 | 69.91 ± 22.76 | 48.85 ± 12.29 |
|  | Non-Responder | 40.68 ± 18.07 | 63.81 ± 28.79 | 72.03 ± 37.21 | 48.94 ± 10.51 |
|  | P-value | 0.461 | 0.153 | 0.637 | 0.981 |
| TSAT (%) | Responder | 11.12 ± 3.72 | 26.25 ± 8.51 | 26.00 ± 8.89 | 21.75 ± 6.80 |
|  | Non-Responder | 12.33 ± 4.30 | 24.98 ± 9.45 | 26.31 ± 11.23 | 26.65 ± 8.94 |
|  | P-value | 0.361 | 0.419 | 0.953 | 0.104 |

TSAT, transferrin saturation
